# Durable responses in patients with HER2+ breast cancer and leptomeningeal metastases treated with trastuzumab deruxtecan

**DOI:** 10.1038/s41523-023-00519-0

**Published:** 2023-03-30

**Authors:** Laura Alder, Dario Trapani, Claire Bradbury, Amanda E. D. Van Swearingen, Sara M. Tolaney, Mustafa Khasraw, Carey K. Anders, Christopher D. Lascola, Liangge Hsu, Nancy U. Lin, Sarah Sammons

**Affiliations:** 1 Division of Medical Oncology, Department of Medicine, Duke Cancer Institute, Duke University, Durham, NC USA; 2 Medical Oncology, Dana-Farber Cancer Institute, Boston, MA USA; 3 Breast Oncology Program, Dana-Farber Brigham Cancer Center, Boston, MA USA; 4 Harvard Medical School, Boston, MA USA; 5 Department of Biostatistics, Duke Cancer Institute, Duke University, Durham, NC USA; 6 Department of Radiology, Duke University, Durham, NC USA; 7 Department of Radiology, Brigham and Women’s Hospital, Boston, MA USA

**Keywords:** Breast cancer, Cancer

## Abstract

Leptomeningeal metastases (LM) are a devastating complication of HER2 + metastatic breast cancer (MBC), with no effective treatments. In a case series of 8 patients with heavily pretreated HER2 + MBC and progressing LM, all 8 patients (100%) derived clinical benefit from Trastuzumab deruxtecan (TDXd), and 4 patients (50%) had an objective partial response based on formal neuroradiology MRI reads using the EORTC/RANO-LM Revised-Scorecard. T-DXd warrants further study in LM in HER2 + MBC and solid tumors where T-DXd may be active.

Between 5–15% of patients with metastatic breast cancer (MBC) develop leptomeningeal metastases (LM)^1,2^ with approximately 60% having no parenchymal brain metastases (BrMs)^3^. The presence of LM has a drastic impact on patients’ function and quality of life, leading to poor survival outcomes, with a median survival of only 4.4 months in human epidermal growth factor receptor 2-positive (HER2+) MBC^4^. Despite significant advances in the treatment of HER2 + MBC in recent years, there are no U.S. Food and Drug Administration (FDA)-approved therapies specifically to treat LM. Trastuzumab deruxtecan (T-DXd) is a HER2-directed antibody-drug conjugate carrying an exatecan-derivative topoisomerase I inhibitor payload^5^.

DESTINYBreast03 demonstrated a significant improvement in progression-free survival (PFS) compared with trastuzumab emtansine (T-DM1), and established a new therapeutic option in the second-line setting^6^. The benefit of T-DXd was observed in the subset of patients with stable BrMs at the time of the enrollment, with an overall response rate (ORR) of 67.4% and BrMs-ORR of 63%^7^. Notably, patients with LM were not eligible in this trial. Smaller studies have evaluated T-DXd to treat active BrMs, showing promising activity. The DEBBRAH and TUXEDO-1 clinical trials, including patients with active (untreated or progressing) BrMs, demonstrated an intracranial ORR of 46.2% (95% CI, 19.2–74.9)^8^ and 73.3% (95% CI, 48.1–89.1%) respectively^9^. Likewise, a recent multi-institution analysis of patients with active HER2 + BrMs showed a brain metastases disease control rate of 75%^10^. The impressive intracranial and extracranial activity of TDXd has led to widespread uptake in the clinic. However, the efficacy of T-DXd in patients with HER2 + LM have not been previously reported.

The assessment of treatment-response of LM in clinical practice and clinical trials remains challenging. Recently, a joint effort of the European Organisation for Research and Treatment of Cancer (EORTC) Brain Tumor Group and Response Assessment in Neuro-Oncology (RANO) prospectively validated a revised magnetic resonance imaging (MRI)-scorecard for response assessment in LM, showing a substantial interobserver agreement and associated prognostic significance^3^.

In this retrospective study inspired by our collective clinical observations, we report the activity of T-DXd in patients with HER2 + MBC and active LM, based on the standardized EORTC/RANO MRI-scorecards. The objective of our study was to assess the activity of T-DXd on LM in patients with HER2 + MBC and progressing LM.

Eight patients met the inclusion criteria at the data cut-off (Table 1). Three patients had baseline, positive cerebrospinal fluid (CSF) cytology, three had negative CSF cytology and two patients were not tested. Median age was 42.5 years; 75% of patients had estrogen receptor-positive MBC. Seven patients (87.5%) had prior whole-brain radiation therapy (WBRT), with a median time of 100 days from the most recent RT to the first infusion of T-DXd (range: 5–363 days). Patients were very heavily pretreated with a median of 4.5 prior therapies (Fig. 1). All had received prior HER2-targeted tyrosine kinase inhibitors (TKI): seven patients had prior tucatinib and one neratinib; two patients had received intra-thecal trastuzumab (Fig. 1). Patients had been alive with active LMD for a median of 101 days prior to T-DXd.

**Table 1:** Demographic Characteristics and Clinical Outcomes.

	Patients (*N* = 8)# (%) or Median (Range)
**Median Age at T-DXd Start**	42.5 (37–56)
**Gender**	
Female	8 (100.0%)
**Race**	
Black	1 (12.5%)
White	7 (87.5%)
**Ethnicity**	
African American	1 (12.5%)
Arab-Middle Eastern	1 (12.5%)
Not Hispanic or Latino	6 (75.0%)
**ER Status**	
Positive	6 (75.0%)
Negative	2 (25.0%)
**HER2 IHC**	
Positive	5 (62.5%)
Negative	3 (37.5%)
**HER2 FISH**	
Positive	5 (62.5%)
Negative	3 (37.5%)
**Status eCNS**	
Absent	5 (62.5%)
Present	3 (37.5%)
**Baseline CSF Cytology**	
Positive	2 (25.0%)
Negative	3 (37.5%)
NA	3 (37.5%)
**Status eCNS 1st Response T-DXd**	
Partial Response	3 (37.5%)
NA	5 (62.5%)
**Site**	
DFCI	5 (62.5%)
Duke	3 (37.5%)
**Median Number Prior Therapies**	4.5 (3–6)
**Prior HER2 TKI**	8 (100.0%)
**Prior Tucatinib**	7 (87.5%)
**Prior Radiation**	7 (87.5%)
**RT Type**	
SRS and WBRT	4 (50.0%)
WBRT	3 (37.5%)
**Median Days C1D1 to last RT**	100 (5–363) 3.3 months
NA	1 (12.5%)
Median Days LM diagnosis to C1D1 T-DXd	101 days (5–309) 3.3 months
**Response on 1**^**st**^ **MRI post-C1D1 T-DXd**	
Partial Response	3 (37.5%)
Stable Disease	5 (62.5%)
NA	0 (0.0%)
**Best Response on MRI post-C1D1 T-DXd**	
Partial Response	4 (50.0%)
Stable Disease	4 (50.0%)
NA	0 (0.0%)
**Clinical Benefit**	8 (100.0%)
**Alive**	6 (75.0%)
**Remains on T-DXd**	5 (62.5%)
**Median Number Cycles T-DXd**	10 (5–23)
**Median Duration T-DXd (days)**	230.5 (127–514) 7.6 months
**Median Survival Since C1D1 T-DXd (days)**	316 (158–514) 10.4 months
**Median Survival Since LM Diagnosis (days)**	471 (189–626) 15.5 months

*T-DXd* Trastuzumab deruxtecan, *ER* estrogen receptor, *HER2* human epidermal growth factor receptor 2, *IHC* immunohistochemistry, *FIS*H fluorescence in situ hybridization, *eCNS* extraCNS, *CSF* cerebral spinal fluid cytology, *DFCI* Dana-Farber Cancer Institute, *TKI* tyrosine kinase inhibitor, *RT* radiation therapy, *SRS* stereotactic radiosurgery, *WBRT* whole brain radiation therapy, *C1D1* Cycle 1 Day 1, *LM* leptomeningeal metastases, *MRI* magnetic resonance imaging.

**Fig. 1:**
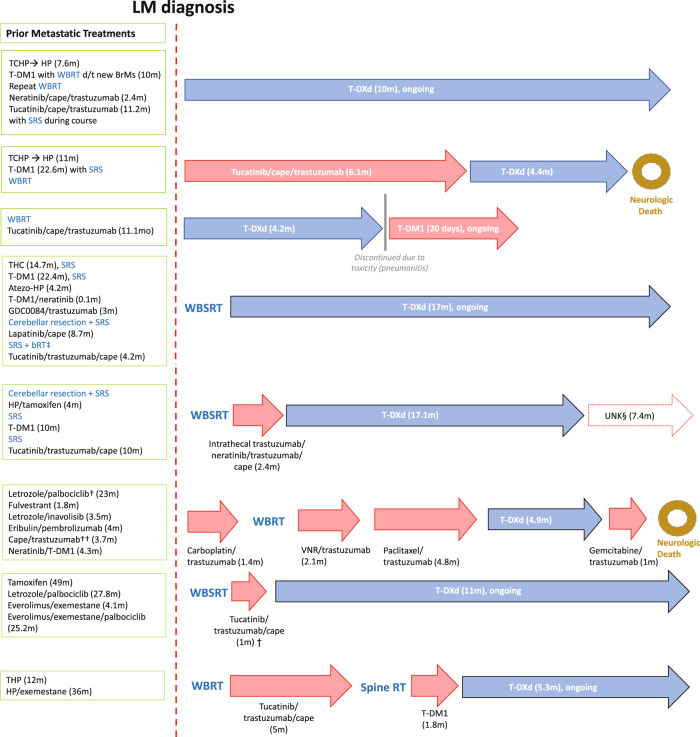
Synoptic presentation of systemic and local treatments from metastatic disease diagnosis to LM course including T-DXd treatment. LM leptomeningeal disease, M months. TCHP trastuzumab, pertuzumab, carboplatin and docetaxel, THC trastuzumab, pertuzumab and docetaxel. HP pertuzumab and trastuzumab. Atezo-HP Atezolizumab, pertuzumab, and high-dose trastuzumab. T-DM1 trastuzumab emtansine. GDC0084 paxalisib. Cape capecitabine. WBSRT whole brain and spine radiotherapy. SRS stereotactic radiation therapy. WBRT whole brain radiation therapy. VNR vinorelbine. T-DXd trastuzumab deruxtecan. BrMs brain metastasis. ‡bRT brachytherapy with radioactive seed implantation. † new biopsy showed HER2 in situ hybridization positive, UNK§, patient was given recommendation for further treatment, lost to follow-up; alive at data cutoff.

Median time on T-DXd was 7.6 months (range:127–514 days) with a median of 10 (5–23) cycles. Five patients remained on T-DXd at the data cut-off (Fig. 2). One half of patients (*n* = 4/8) achieved a partial response (PR) on MRI as best response based on the EORTC/RANO-LM Revised-Scorecard, while 4 patients had stable disease (SD) (Fig. 3). Clinical benefit response (CBR) was 100%. Of note, two patients with RANO-LM defined as SD did show radiographic improvement of leptomeningeal linear enhancement, which is not considered evaluable for response. No patient experienced BrMs progression while on T-DXd. No patient had available post-baseline CSF cytology analysis. The median overall survival (OS) from the first cycle of T-DXd was 10.4 months (158–514 days) with 6 patients still alive at the data cut-off. Both deceased patients died due to neurological deterioration. The median OS from the diagnosis of LM was 15.5 months (189–626 days).

**Fig. 2:**
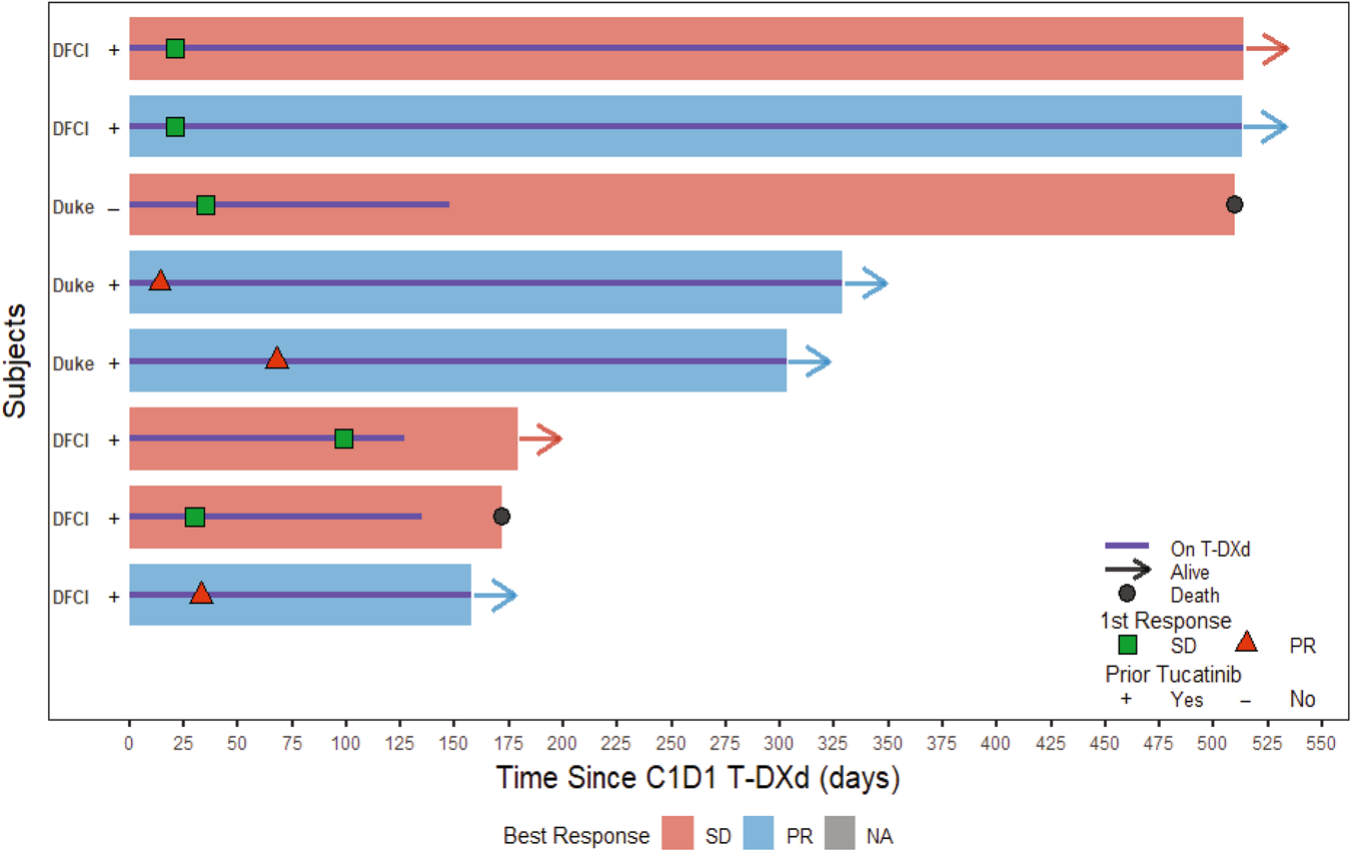
Overall survival and time on T-DXd in a series of 8 patients with breast cancer HER2 + leptomeningeal metastases. Swimmers plot depicting time on treatment, best response by formal neuroradiology review and overall survival in patients with HER2+ LM treated with T-DXd.

**Fig. 3:**
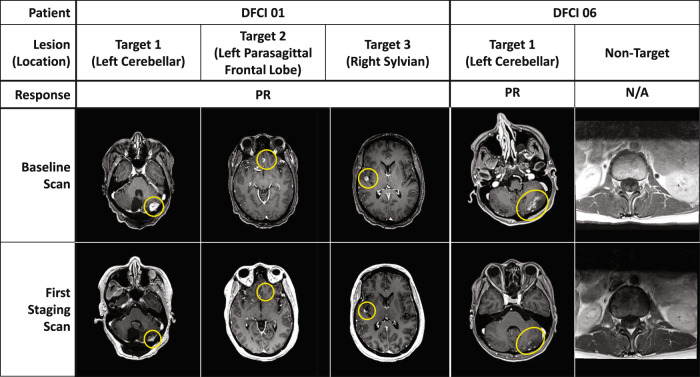
Radiographic responses to progressive HER2 + LM treated with T-DXd. Baseline and LM response assessment by MRI Brain imaging of two patients with HER2+ LM treated with T-DXd. Encircled are unirradiated CNS lesions highlighting response.

In our case series, T-DXd demonstrated encouraging activity with an LM ORR of 50% and CBR of 100%. The median time on treatment was 7.6 months with 5 patients ongoing at the data cutoff. The median OS was 10.4 months since the start of T-DXd with 6 patients remaining alive at the data cutoff. Of note, the median OS from the diagnosis of LM was 15.5 months, suggesting that patients have derived most of the benefit from T-DXd, since the diagnosis of LM. Patients were heavily pretreated, all having received prior RT and a median of 4.5 prior lines of systemic therapy in the metastatic setting, including tucatinib. Limited therapeutic options exist for such patients. In general, LM spread represents a critical event for patients with MBC, with significant prognostic implications, mostly related to the lack of high-efficacy treatments demonstrating durable responses. The major challenge pertains to the CSF-widespread nature of the LM seeding, and the need to treat the whole disease burden. Traditionally, patients with LM have been managed with RT for bulky symptomatic disease with emerging evidence for cranio-spinal RT followed by systemic and/or intrathecal therapies. Attempts for effective intra-CSF drug delivery have been provided through high-dose systemic chemotherapy regimens, like methotrexate, or directly infused in the CSF via lumbar puncture or ventricular reservoir, most commonly methotrexate, liposomal cytarabine and thiotepa, resulting in 30–50% ORR but limited survival rates of approximately 3–5 months^11,12^. Intrathecal administration of therapies comes with substantial risk and is clinically challenging.

Only select anti-HER2 therapies have reported activity in HER2 + LM. In particular, neratinib plus capecitabine showed preliminary activity in LM, with median LM-PFS of 4 months and OS of 10 months^13^. In addition, the use of intrathecal trastuzumab has demonstrated ORR of 19.2%, SD in 50%, with a CBR of 70%, and median-OS of 10.5 months^14^. Given the favorable CSF pharmacokinetic profile and proven intra-cranial activity, tucatinib has been identified as a potential therapeutic for patients with LM, and is under investigation in the TBCRC049 trial (NCT03501979)^15^. Until now, antibody-drug conjugates held little promise for intracranial efficacy. The body of evidence for TDXd efficacy in stable and active HER2 + BrMs has challenged this paradigm. A potential advantage of T-DXd is the possibility of activity in HER2-low LM in patients with breast cancer, and even potential for activity in LM from other solid tumors, given the broad extracranial efficacy profile reported to date.

Major limitations of our study include its retrospective nature and small sample size, both of which carry intrinsic risks of bias. Also, baseline CSF cytology was rarely available and not performed serially, and LM diagnosis was mostly based on MRI. The centralized neuroradiology assessment of the response based on validated methodology is an important strength of this analysis. Prospective clinical trials are rarely funded in patients with LM and new ways of obtaining clinical data such as prospective registries and multi-institutional case series are needed.

In conclusion, we report activity in a case series of heavily pre-treated progressing HER2 + LM patients treated with TDXd,. Prospective clinical trials are warranted.

## Methods

### Patient Characteristics

Using institutional electronic medical records, our doctors identified patients with metastatic HER2+ breast cancer at Duke Cancer Institute (Duke) and Dana-Farber Cancer Institute (DFCI) who had brain and/or spine MRI-confirmed LM (+/− positive cytology) and who had received at least one dose of T-DxD between January 1, 2020 and December 31, 2020 as part of their usual clinical care. Data on demographics, clinical, pathologic, and treatment related to their primary and MBC diagnoses were also abstracted based on medical record review.

58 patients treated with T-DXd at Duke and DFCI were identified in the timeframe with biopsy-proven HER2 + metastatic breast cancer. (30 patients at Duke – and 28 patients at DFCI). Amongst those patients, 8 had evidence of progressing LM by clinical assessment, MRI imaging+/− cytology. We utilized the European Association of Neuro-Oncology/European Society for Medical Oncology (EANO/ESMO) proposed classification of LM from solid cancers based on clinical, MRI, and CSF cytology presentation in identification of LMD^16^. Patients with Type I LM defined by positive CSF cytology (confirmed LM) and type II LM defined by typical clinical and MRI signs (probable or possible LM) were included. As is the case in clinical practice, not all patients underwent CSF testing.

### Data collection and evaluation

Retrospective data collection included patients’ demographic and tumor clinicopathologic characteristics, previous RT, presence of BrMs, and previous anti-HER2 therapies.

Radiological assessment was based on the EORTC/RANO revised LM scorecard^3^ and performed by a dedicated neuroradiologist at DFCI (HL) and Duke (CL). The scorecards were completed for each patient at each MRI re-evaluation; BrMs response was also reported (Supplementary Table 1). LM partial response corresponds to a decrease by >50% in the summed product of orthogonal diameters of the LM implants, with no increase of the ventricular size. ORR included patients reporting a PR or complete response (CR). CBR included the proportion of patients experiencing SD, PR, or CR, with stable or improved neurological symptoms. Available reports on CSF analyses were all collected.

## Supplementary information

Supplementary Table 1

## Data Availability

The datasets of this study are hosted with a protected password known to the lead authors in a data repository and are not for open access, given their sensitive nature. The corresponding author, Sarah Sammons, MD (sarahl_sammons@dfci.harvard.edu) may be contacted for potential collaborations, upon careful screening of the proposals, to share the data grouped and anonymized, provided an IRB authorization.
