# Supplementary material for: Durable responses in patients with HER2+ breast cancer and leptomeningeal metastases treated with trastuzumab deruxtecan
Source: NPJ Breast Cancer. 2023 Mar 30;9:19. doi: 10.1038/s41523-023-00519-0 (PMC10063529; doi:10.1038/s41523-023-00519-0)
Supplement: Supplementary file 1 — Supplementary Table 1 [file 41523_2023_519_MOESM1_ESM.pdf]

Supplementary Table 1:Radiological assessment of the leptomeningeal disease based on the EORTC-RANO scorecards

<table>
<tr><th>Patient ID</th><th>1° staging</th><th>2° staging</th><th>Last staging *</th><th>Comment</th></tr>
<tr><td>DF01</td><td>LMD:

-Brain
• N1: -19%
• N2: -61%
• N3: -59%

-Spine: no

-Evan's index: ratio = 0.97
-Hydrocephalus: no

Brain mets: no

<u>Overall response</u>: SD</td><td>LMD:

-Brain
• N1: -73%
• N2: -90%
• N3: -65%

-Spine: no

-Evan's index: ratio = 1.1
-Hydrocephalus: no

Brain mets: no

<u>Overall response</u>: PR</td><td>LMD:

-Brain
• N1: -4.8%
• N2: -16%
• N3: +300%
• N4 (new lesion)

-Spine: no

-Evan's index: ratio = 0.95
-Hydrocephalus: no

Brain mets: no

<u>Overall response</u>: PD</td><td>PD confirmed with the subsequent MRI in 4/2021.</td></tr>
<tr><td>DF03</td><td>LMD:

-Brain
• N1: -59%
• N2: -44%
• N3: -41%

-Spine: no

-Evan's index: ratio = 1
-Hydrocephalus: no

Brain mets: no

<u>Overall response</u>: SD</td><td>LMD:

-Brain
• N1: -37%
• N2: -20%
• N3: -53%

-Spine: no

-Evan's index: ratio = 0.97
-Hydrocephalus: no

Brain mets: no

<u>Overall response</u>: SD</td><td>LMD:

-Brain
• N1: -48%
• N2: -100%
• N3: -100%

-Spine: no

-Evan's index: ratio = 0.94
-Hydrocephalus: no

Brain mets: no

<u>Overall response</u>: SD</td><td>Discontinued all treatments a month later, for extra-cerebral PD, and rapid clinical deterioration with fatal outcome.</td></tr>
</table>

Patient ID	1° staging	2° staging	Last staging *	Comment
**DF05**	LMD:<br><br>-Brain<br>• N1: -59.3%<br>• N2: non-measurable, present<br><br>-Spine: no<br><br>-Evan's index: ratio = 0.94<br>-Hydrocephalus: no<br><br>Brain mets: no<br><br><u>Overall response</u>: PR	LMD:<br><br>-Brain<br>• N1: -37%<br>• N2: non-measurable, present<br><br>-Spine: no<br><br>-Evan's index: ratio = 1<br>-Hydrocephalus: no<br><br>Brain mets: no<br><br><u>Overall response</u>: SD	LMD:<br><br>-Brain<br>• N1: -100%<br>• N2: non-measurable, present<br><br>-Spine: no<br><br>-Evan's index: ratio = 1.05<br>-Hydrocephalus: no<br><br>Brain mets: no<br><br><u>Overall response</u>: PR	
**DF06**	LMD:<br><br>-Brain<br>• N1: -56%<br><br>-Spine: diffuse, cauda equina, non-measurable, present<br><br>-Evan's index: ratio = 1.02<br>-Hydrocephalus: no<br><br>Brain mets: no<br><br><u>Overall response</u>: PR	LMD:<br><br>-Brain<br>• N1: -69.5%<br><br>-Spine: diffuse, cauda equina, non-measurable, present<br><br>-Evan's index: ratio = 1<br>-Hydrocephalus: no<br><br>Brain mets: no<br><br><u>Overall response</u>: PR	The last MRI scan is the second one.	

Patient ID	1° staging	2° staging	Last staging *	Comment
**Duke 1**	LMD:<br><br>-Brain<br>• N1: -55.8%<br><br>-Spine: no<br><br>-Evan's index: ratio = 1.02<br>-Hydrocephalus: no<br><br>Brain mets:<br>• N1: -60.64<br>• N2: -60.9<br><br><u>Overall response</u>: PR	LMD:<br><br>-Brain<br>• N1: -9.76%<br><br>-Spine: no<br><br>-Evan's index: ratio = 1.02<br>-Hydrocephalus: no<br><br>Brain mets:<br>• N1: -100<br>• N2: -25.7<br><br><u>Overall response</u>: PR	MD:<br><br>-Brain<br>• N1: -100<br><br>-Spine: no<br><br>-Evan's index: ratio = 0.972<br>-Hydrocephalus: no<br><br>Brain mets: no<br>• N1: N/A<br>• N2: -63.9<br><br><u>Overall response</u>: PR	
**Duke 2**	LMD:<br><br>-Brain<br>• N1: -75.0%<br>• N2: -51.5%<br>• N3: -41.61%<br><br>-Spine:<br>N1: -59.5<br><br>-Evan's index: ratio = 0.97<br>-Hydrocephalus: no<br><br>Brain mets: no<br><br><u>Overall response</u>: SD			subependymal and conus response; otherwise, no response

Patient ID	1° staging	2° staging	Last staging *	Comment
**Duke 3**	LMD:<br><br>-Brain: nodular and linear enhancement within the cerebellar folia, non-measurable, present, decreased<br><br>-Spine: Leptomeningeal carcinomatosis, non-measurable, present, decreased<br><br>-Evan's index: ratio = 0.95<br><br>-Hydrocephalus: no<br>Brain mets: no<br><br><u>Overall response</u>: SD	LMD:<br><br>-Brain: nodular and linear enhancement within the cerebellar folia, non-measurable, present, further decreased<br><br>-Spine: Leptomeningeal carcinomatosis, non- measurable, present, further decreased<br><br>-Evan's index: ratio = 1.029<br><br>-Hydrocephalus: no<br>Brain mets: no<br><br><u>Overall response</u>: SD		1st scan: decreased linear LMD in brain and spine, not measurable<br><br>2nd scan: further decreased linear enhancement, nothing measurable)

All disease response assessments are based on two consecutive MRI scans, as per RANO methodology (Le Rhun, 2022).
PD, disease progression. PR, partial response. SD, stable disease. LMD, leptomeningeal disease.

* up to March 31, 2022, <u>as compared with the penultimate MRI scan</u> (that could be not the same as the 2nd disease re-assessment).
